# Snacking Quality Is Associated with Secondary School Academic Achievement and the Intention to Enroll in Higher Education: A Cross-Sectional Study in Adolescents from Santiago, Chile

**DOI:** 10.3390/nu9050433

**Published:** 2017-04-27

**Authors:** Paulina Correa-Burrows, Yanina Rodríguez, Estela Blanco, Sheila Gahagan, Raquel Burrows

**Affiliations:** 1 Institute of Nutrition and Food Technology, University of Chile, Santiago 7830490, Chile; yanirod77@hotmail.com (Y.R.); rburrows@inta.uchile.cl (R.B.); 2 Division of Child Development and Community Health, University of California, San Diego, CA 92093, USA; esblanco@ucsd.edu (E.B.); sgahagan@ucsd.edu (S.G.)

**Keywords:** adolescents, unhealthy eating, snacks, academic performance, diet quality

## Abstract

Although numerous studies have approached the effects of exposure to a Western diet (WD) on academic outcomes, very few have focused on foods consumed during snack times. We explored whether there is a link between nutritious snacking habits and academic achievement in high school (HS) students from Santiago, Chile. We conducted a cross-sectional study with 678 adolescents. The nutritional quality of snacks consumed by 16-year-old was assessed using a validated food frequency questionnaire. The academic outcomes measured were HS grade point average (GPA), the likelihood of HS completion, and the likelihood of taking college entrance exams. A multivariate analysis was performed to determine the independent associations of nutritious snacking with having completed HS and having taken college entrance exams. An analysis of covariance (ANCOVA) estimated the differences in GPA by the quality of snacks. Compared to students with healthy in-home snacking behaviors, adolescents having unhealthy in-home snacks had significantly lower GPAs (*M* difference: −40.1 points, 95% confidence interval (CI): −59.2, −16.9, *d* = 0.41), significantly lower odds of HS completion (adjusted odds ratio (aOR): 0.47; 95% CI: 0.25–0.88), and significantly lower odds of taking college entrance exams (aOR: 0.53; 95% CI: 0.31–0.88). Unhealthy at-school snacking showed similar associations with the outcome variables. Poor nutritional quality snacking at school and at home was associated with poor secondary school academic achievement and the intention to enroll in higher education.

## 1. Introduction

In spite of efforts by public agencies to monitor the types of food sold in school settings or regulate food advertising aimed at young people, their exposure to energy-dense foods (those with a high caloric concentration per bite) at and away from school remains high [1]. A recent study on the consumption of fast food in 36 developed and developing countries showed that more than 50% of adolescents consume fast food frequently or very frequently [2]. In Latin America, the Global School-based Health Survey (GSHS) showed that two-thirds of adolescents (13–17 years old) in Argentina, Chile and Uruguay reported daily intake of sugar-sweetened beverages [3]. In the early 2010s, among European 15-year-old, daily soft drink consumption was more than 40% in England, the Netherlands, Belgium, Slovakia and Slovenia [4].

Evidence is available on the role of Western-type diets (WD) in limiting cognitive abilities in critical brain maturation periods (i.e., infancy and childhood) [5,6]. Animal models show that exposure to a high-fat, high-sugar (HFS) diet in adolescence is related to impairment in hippocampal learning and memory processes, regardless of weight status [7,8]. One important mechanism that is proposed to underlie HFS-induced impaired hippocampal function is the reduced synthesis, secretion, and action of the brain-derived neurotrophic factor (BDNF). BDNF facilitates synaptic efficacy by converting changes in electrical activity to long-lasting changes in synaptic function, which is a suggested key process for memory formation [9]. Reduced levels of BDNF in association with impaired memory function has been well documented in the literature [10,11].

Impairment of memory consolidation and memory performance is a risk factor for learning difficulties and poor academic progress [12]. Thus, a diet of poor nutritional value may compromise students’ ability to perform well in school. Longitudinal and cross-sectional studies, mostly conducted in developed countries, have examined the relationship between diet and school grades [13,14,15], as well as the relationship between diet and performance on standardized academic tests [16,17,18]. Results collectively suggest that better educational outcomes are associated with regular consumption of nutritious breakfasts, lower intake of energy-dense, nutrient-poor foods, and maintaining a healthy diet [19].

The effect of WDs on academic results can be used to strengthen health promotion strategies. While the connection between unhealthy diet and poor academic performance (as measured by school grades and standardized test scores) in elementary and middle schoolers has been well described, less is known about the relationship between dietary habits and postsecondary educational aspirations—that is, the intention to pursue higher education after secondary school. The increasing number of HS graduates seeking entrance to higher education institutions, including in non-industrialized nations, has made this a particularly important topic for students, families and policymakers.

Since the question of how WD foods may compromise students’ intention to pursue higher education is also of interest to non-academic audiences, we used a translational-research approach to provide evidence that can be translated from research and applied to practice and policy. Thus, we examined the relationship of nutritional quality of snacks with academic outcomes using functional cognition measures like grade point average (GPA), high school (HS) completion, and college entrance examination participation rates. Our decision to concentrate on snacks rather than overall diet or meals such as breakfast, lunch or supper was based on wanting to focus on food choices made by adolescents rather than consumption of foods over which they may have little volition. We hypothesized that students habitually eating unhealthy snacks would have lower grades and be less likely to complete HS and take college admission exams.

## 2. Materials and Methods

### 2.1. Study Design and Population

We studied 16–17-year-old adolescents living in Santiago, Chile, from low-to-middle socioeconomic status (SES), who were part of an infancy cohort. Participants were recruited at 4 months from public healthcare facilities in the southeast area of Santiago (*n* = 1791). They were born at term of uncomplicated vaginal births, weighed >3.0 kg, and were free of acute or chronic health problems. At 6 months, infants free of iron deficiency anemia (*n* = 1657) were randomly assigned to receive iron supplementation or no added iron (ages 6–12 months). They were assessed for developmental outcomes in infancy, and at 5, 10 and 15 years [20]. At 16–17 years, those with complete data in each wave (*n* = 678) were also assessed for obesity risk and the presence of cardiovascular risk factors in a half-day evaluation that included assessment of dietary habits and nutritional content of food intake. Ethical approval was obtained by the institutional review boards of the University of Michigan, Institute of Nutrition and Food Technology (INTA), University of Chile, and the University of California, San Diego. Participants and their primary caregiver provided informed and written consent, according to the norms for Human Experimentation, Code of Ethics of the World Medical Association (Declaration of Helsinki, 1995).

### 2.2. Nutritional Quality of Snacking at Age 16

Nutritional quality of in-school and at-home snacking was measured considering the amount of saturated fat, fiber, sugar and salt in the food. Assessment was performed with a food frequency questionnaire, validated using three 24 h recalls to include weekends [21,22]. A section of this questionnaire was specially designed to assess the usual diet during the snack time at school and at home, by asking about the frequency of food consumption within the past three months. A list of 50 foods and beverages was used. The frequency of food consumption was assessed by a multiple response grid; respondents were asked to estimate how often a particular food or beverage was consumed. Categories ranged from ”never” to ”five or more times a week”. The electronic version of the Chilean Food Composition Tables/Database was used to assess the quality of snacks composition [23]. Food items were classified as unhealthy (poor nutritional value items, high in fat, sugar, salt and calories), unhealthy-to-fair (highly processed items although low in fat) and healthy (nutrient rich foods). We assigned adjustment weights to each food item conditioned to its nutritional quality. A score ranging from 0–10 was computed by adjusting the frequency of food consumption to the nutritional quality of foods consumed during the snack time. For each snacking type (in-school or at-home), participants had a continuous score, with higher scores representing healthier snacking habits. We applied quartile cutoffs for the Chilean adolescent population (comprising students of high-, middle- and low-SES) to classify the nutritional quality of in-home and at-school snacking of participants into three groups: unhealthy (≤4.3 or ≤25th percentile), unhealthy-to-fair (from 4.4 to 5.9 or >25th percentile and <75th percentile) and healthy (≥6.0 or ≥75th percentile) [21].

### 2.3. Academic Outcomes

The academic outcomes measured were HS GPA, the likelihood of HS completion, and the likelihood of taking college entrance exams. Data on GPA and high school completion were obtained from publicly available records at the Academic Assessment Unit of the Ministry of Education of Chile. Following the Ministry of Education criteria, GPA (on a scale of 1–7) was transformed into standardized scores (ranging from 210–825), and adjusted by type of secondary education (academic, vocational or adult school). Data on college examination rates were derived from publicly available information from the Assessment and Measurement Department of the University of Chile, which administers the tests for college entrance on behalf of the Ministry of Education. Although the exams for college admission are non-mandatory for HS graduates (only for those aiming at enrolling in higher education), more than 85% of Chilean HS graduates take the tests and, thus, have plans for future schooling [24].

### 2.4. Weight Status at Age 16

A research physician used standardized procedures to measure the adolescent’s height (cm) and weight (kg) in duplicate. Body mass index (BMI = kg/m^2^) at age 16 was evaluated and z-scores were estimated according to the World Health Organization (WHO) 2007 references [25]. Weight status was defined as follows: underweight (BMI-z < −1 SD), normal weight (BMI-z from −1 SD to 1 SD), overweight (BMI-z from 1 SD to <2 SD) and obesity (BMI-z ≥ 2 SD).

### 2.5. Physical Activity at Age 16

Physical activity has been found to be associated with academic achievement in studies conducted in Chile [26,27]; therefore, it could be a relevant confounder for the association between diet and academic results. We approached physical activity habits with scheduled, repetitive and planned exercise, accounting for the number of weekly hours devoted to Physical Education (PE), and extracurricular sports. To measure this, we used a questionnaire that was validated in a previous study using accelerometry-based activity monitors in both elementary and high school children [28]. The questionnaire was administered by a researcher to all students at the time they attended the anthropometric examination. Participants were asked: (1) On average, over the past week, how often did you engage in PE? (2) On average, over the past week, how often did you engage in extracurricular sports, either school- or non-school-organized? (3) On those days, on average, how long did you engage in such activities? With this information, we estimated the average hours per week of scheduled physical activity. Participants having ≤90 min of weekly scheduled physical activity, which is the mandatory time for school-based PE, were considered to be physically inactive.

### 2.6. Other Covariates Collected in Previous Waves

Parental educational attainment is an important measure of human capital level among populations and, also, is an important predictor of children’s educational outcomes [29]. In infancy, participant’s mother and father were asked to report the highest schooling level they have been enrolled in, as well as the highest grade they completed at that level. In our analysis, five standard hierarchic levels were defined according to the 2011 International Standard Classification of Education: (1) no education completed; (2) first level (primary school or 1st–8th); (3) secondary level (first phase or 9th–10th); (4) secondary level (second phase or 11th–12th); and (5) post-secondary non-tertiary educations or short-cycle tertiary education [30]. Then, we merged these categories into two: incomplete secondary education (1 + 2 + 3), and complete secondary education or higher (4 + 5). In health research, parental education has been often used as proxy for socioeconomic background [31]. Also, because the literature describes correlations between children’s educational outcomes and family structure [32], we include a variable denoting whether the participant was raised in a fatherless family. This information was reported by the participant’s parents or guardian. Finally, to control potential design biases, we used a categorical variable denoting whether the participant had received iron supplementation or no added iron at 6–12 months.

### 2.7. Statistical Analysis

Data were processed using Stata SE for Windows 12.0 (Lakeway Drive College Station, TX, USA). All categorical data were expressed as absolute and relative frequencies, while continuous data were expressed as means and standard deviations. Statistical analysis included χ^2^ for categorical variables, and analysis of variance (ANOVA) with Bonferroni correction for comparison of means. We tested for effect measure modification (interaction) by weight status and physical activity, in the association between quality of snacking and academic outcomes using two-way ANOVA. The interaction of quality of snacking with weight status and physical activity was non-significant at *p* < 0.05 and, therefore, we did not stratify the analysis. Unadjusted logistic models were used to explore cross-sectional patterns of variation in academic behavior across snack categories (unhealthy and unhealthy-to-fair vs. healthy). Next, the models were adjusted for sex, weight status, physical activity, familial background and a variable to control potential design biases. Odds ratios are presented in the tables with 95% CI to evaluate the strength and precision of the associations. Analysis of covariance (ANCOVA) was used to determine whether high school GPA differed by nutritional quality of snacking, accounting for the same potential confounders. Because GPA scores do not have an intrinsic meaning, the effect size for difference was estimated using Cohen’s *d* coefficients. A *p* < 0.05 denoted statistical significance.

## 3. Results

As shown in Table 1, our sample was composed of 16.8-year-old (0.3 SD) adolescents (47% males). Eighty-four percent completed HS (*n* = 571) and were allowed to take the exams for college admission. Of them, 68% (*n* = 388) took the college entrance exam. High school GPA ranged from 269–795 points, and mean value was 481.1 (92.3 SD) points. Mean value of BMI-z was 0.65 (1.2 SD). Of the participants, 25% and 14% were overweight and obese, respectively. In the sample, 60% were physically inactive.

**Figure 1:**
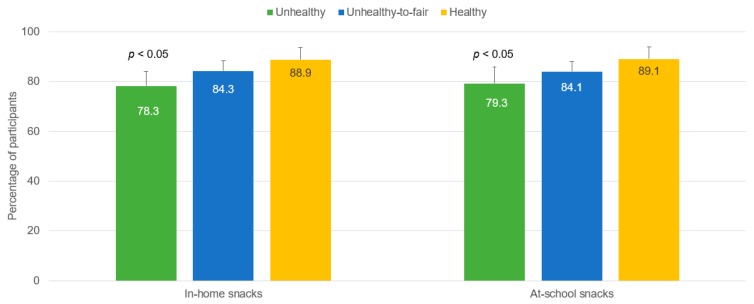
Proportion of students getting their high school diploma (outcome) by nutritional quality of in-home and at-school snacking (exposure) (*n* = 678). Error bars are 95% CI (upper limit). CI: confidence interval.

**Figure 2:**
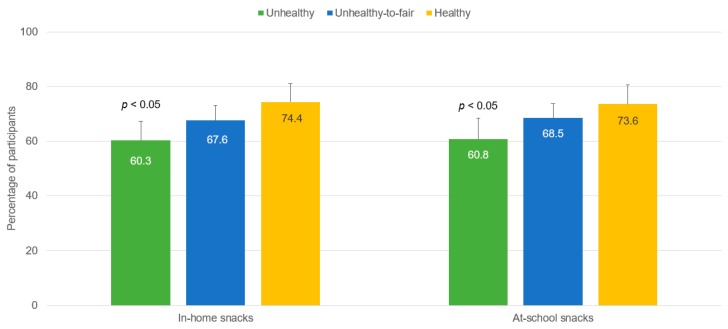
Proportion of participants taking the exams for college admission (outcome) by nutritional quality of in-home and at-school snacking (exposure) (*n* = 571). Only those students graduating from high school (*n* = 571) are allowed to take the exams for college admission. Error bars are 95% CI (upper limit).

The share of students completing the secondary education significantly increased with better nutritional quality of at-school (χ^2^ = 6.73, *p* < 0.05) and in-home (χ^2^ = 7.19, *p* < 0.05) snacking (Figure 1). Likewise, the proportion of students taking the exams for higher education was significantly higher among participants having healthy in-home (χ^2^ = 12.40, *p* < 0.01) and at-school (χ^2^ = 11.66, *p* < 0.01) snacking (Figure 2).

Table 2 shows the estimated cross-sectional association between graduating HS and the nutritional quality of in-home and at-school snacking. After adjusting for sex, weight status, physical activity, parental education, family structure and iron supplementation in infancy, unhealthy snacking significantly reduced the odds of completing the secondary education. For instance, students having unhealthy in-home snacks were 53% (odds ratio (OR): 0.47, 95% CI: 0.25–0.88) less likely to complete HS than students having healthy in-home snacks. Odds were lower but non-significant among students eating foods of unhealthy-to-fair nutritional quality at home compared to those eating healthy snacks at home. When school snacking was the exposure, we also found a positive significant association of nutritional quality of snacks with the likelihood of getting the HS diploma (aOR: 0.49, 95% CI: 0.27–0.89). In all these models, sex and physical activity were also related to the chances of HS graduation.

Similarly, among students who completed HS, the odds of taking the college entrance exam were significantly lower for those having unhealthy in-home snacks compared to those having healthy in-home snacks (Table 3). After controlling other influences, students who reported consumption of unhealthy in-home snacks were 47% less likely (aOR: 0.53; 95% CI: 0.31–0.88) to take the college entrance exam, compared to students eating healthy snack items. In addition, students eating in-home snacks of unhealthy-to-fair nutritional quality had lower odds of taking the college entrance exam, compared to those with healthier habits, though the association was non-significant. When school snacking was the exposure, the odds of taking the examination for college were also lower in students eating unhealthy snacks (aOR: 0.57; 95% CI: 0.35–0.90) compared to those eating healthy at-school snacks. In all these models, the odds of taking the college entrance exam were significantly associated with sex, maternal education and family structure.

**Figure 3:**
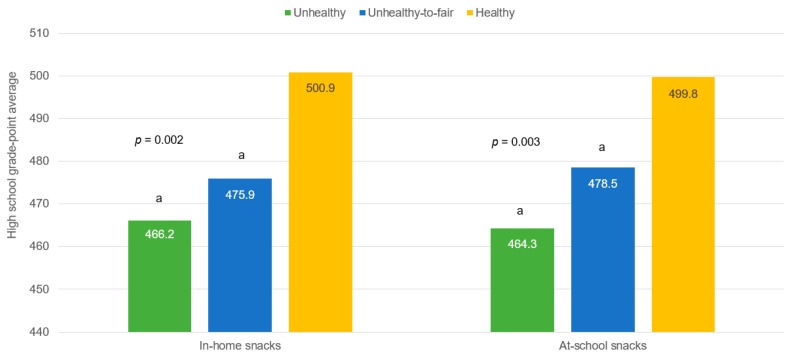
Mean high school grade point average (GPA) by nutritional quality of in-home and at-school snacking (*n* = 571). GPA expressed as standardized score, according to the Chilean Ministry of Education. a, significantly different from the group having healthy snacks at home or at school. *p* value estimated with Analysis of Variance (ANOVA) with Bonferroni adjustment.

The nutritional quality of snacking was also significantly related with students’ final GPA as shown in Figure 3. After accounting for the effect of sex, weight status, physical activity, parental education, family structure and iron supplementation in infancy (Table 4), the group snacking on unhealthy foods at home had a final GPA of 490.0 points, on average, whereas participants having healthy snacks at home had a final GPA of 530.1 points (GPA mean difference = −40.1 points; 95% CI: −59.2; −16.9, *d* = 0.43). When comparing those having unhealthy-to-fair snacks vs. those having healthy snacks at home the GPA mean difference was −27.9 points (95% CI: −43.5; −8.2, *d* = 0.30). It is worth noting that Cohen’s *d* coefficients around 0.20 are considered of interest in educational research when they are based on measures of academic achievement [33]. Lastly, the same pattern was observed when the main exposure was the nutritional quality of at-school snacking.

## 4. Discussion

### 4.1. Main Findings

This study explored whether the nutritional quality of in-home and at-school snacking among high school students in Santiago, Chile, was cross-sectionally associated with secondary school academic achievement and the intention to enroll in higher education. Although numerous studies have approached the effects of short-term exposure to a WD on academic outcomes [19], very few have focused on foods consumed during snack times. We found that unhealthy snacking was correlated with lower high school GPA and rate of graduation, as well as a reduced likelihood of taking college admission exams. When controls for sex and other potentially confounding variables (e.g., weight status, physical activity, familial background, etc.) were entered into the models, unhealthy snacking continued to be associated with worse academic results.

Our findings are consistent with previous research that found evidence of a relationship between a healthy diet and academic achievement. The results of a population-based study of 4th and 8th grade Chilean school children—students from subsidized, partially subsidized, and private schools—showed a positive cross-sectional association between performance in language and mathematics as measured by Chile’s standardized System for the Assessment of Educational Quality test and the nutritional quality of school snacking, regardless of sex, SES, and other educational influences [17]. Similarly, in a subset (*n* = 395) of the current sample, Correa et al. [18] observed that, among students taking college entrance exams, unhealthy dietary habits of 16-year-old were associated with lower performance on college examination tests when compared to the performance of students with healthy dietary habits.

Cross-sectional studies conducted in adolescents from other countries also found that participants having healthy dietary habits performed better at school compared to those having unhealthy dietary habits. For instance, the native and foreign language attainment among 14- and 15-year-old Icelandic students, as well as their mathematics achievement, were negatively influenced by poor dietary habits [13,15]. Norwegian 9th and 10th graders with a high intake of sugar-sweetened soft drinks, candies, chocolate, chips, pizza, hot dogs, and hamburgers were up to 6 times more likely to manifest learning difficulties in mathematics. Conversely, a diet of fresh fruits at least once daily reduced the chances of difficulties in these areas [14]. Also in 15- to 17-year-old Norwegian adolescents, high academic achievement was associated with a high intake of fruits and berries, and a low intake of sugar-sweetened beverages [34]. Unfavorable academic performance, as measured by a standardized test, was positively associated with unhealthy dietary patterns in 6- to 13-year-old Taiwanese students. The likelihood of underperforming on the test was 1.63 times higher for students with greater consumption of low-quality foods (e.g., sweets and fried foods) than it was for students with low intake of such items. Fu et al. also showed that students with poor academic performance were less likely to regularly eat foods that are rich in protein, vitamins and minerals [35].

Diet is also an important influence on other determinants of academic success. Among adolescent students from Iceland, having an optimal diet was cross-sectionally associated with decreased odds of behavioral problems in the classroom [36]. Likewise, in 15- and 16-year-old male students from Oslo (Norway), intake of >4 glasses/day of sugar-sweetened soft drinks more than doubled the probability of having behavioral problems at school, compared to students drinking <1 glass of sugary drinks per day [37]. Among female students in Oslo with excessive intake of sugar-sweetened soft drinks, the chances of conduct problems at school were 4.1 times higher compared to the reference group. In males, soft drink consumption was also related to hyperactivity and higher levels of mental distress, both of which are associated with academic difficulties [38].

It is likely that the effect on academic results of excessive consumption of foods high in saturated fats and simple sugars is mediated by the effect of these macronutrients on brain health and cognitive function. In developmental stages such as adolescence, the brain is particularly vulnerable to the effects of excessive intake of saturated fats and simple carbohydrates [5,6]. Diet-induced impairment in learning and hippocampus-dependent memory processes have been widely documented [7,8,39,40]. In addition to reducing production of neurotrophins such as BDNF, other WD-induced effects have been reported on this brain structure, including overexpression of proinflammatory cytokines, mitochondrial damage due to oxidative stress, and altered blood–brain barrier permeability [7,8,10,39]. Also, insulin resistance and hyperleptinemia have been linked to impaired hippocampal synaptic plasticity and poor cognitive functioning [41,42,43]. Furthermore, evidence suggests that juvenile exposure to a WD may be more harmful than such exposure in adulthood. A 3-week juvenile WD regimen induced similar weight gain and metabolic alterations as did a 12-week adult WD regimen. Juvenile exposure, however, also affected memory consolidation and flexible memory expression while promoting exaggerated pro-inflammatory cytokine expression in the hippocampus after an immune challenge, and it diminished hippocampal neurogenesis [8,39].

While the cross-sectional design of our study prevents definitive conclusions about causality and the direction of the associations depicted here, it is worth noting that research conducted in both animals and humans described short-term effects of Western-type dietary habits on hippocampal-dependent learning and memory. In animals, it is well established that a WD causes rapid impairments of hippocampal-based tasks, with diet-related cognitive effects observed after only 72 h [44,45]. Studies in humans are limited but they confirm that a WD impacts hippocampal memory tasks following a relatively short exposure. Healthy 20-year-old college students from Australia consuming a HFS breakfast (30% saturated fats plus 18% refined sugars), over four consecutive days, showed significantly poorer memory recall compared to control students consuming a healthier breakfast of similar palatability and food types, but significantly lower in saturated fats and refined sugars (5% saturated fats plus 10% sugars). Since these changes in memory performance were linked to shifts in blood glucose across breakfast, authors suggest that this could be one potential mechanism by which a WD affects hippocampal function [46]. In a similar manner, in sedentary men aged 25–45 years, Edwards et al. found decreased power of attention and increased simple reaction time after seven days of consuming a diet comprising 74% kcal. from fat [47]. It is less clear for how long the cognitive effects of a WD will remain and, thus, further investigations should address that question. Although experimental studies in humans show that improvements in memory can occur following reductions in energy intake and fat [48], or shift to a diet low in saturated fats and refined sugars [49], observational longitudinal studies conducted in Anglo-Saxon countries suggest that unhealthy dietary practices in developmental periods have a lasting association with cognitive and educational outcomes that seem to persist over time, regardless of later changes in diet [16,50,51,52].

Our results also showed that a significant share (73%) of participants in the sample ate snacks of intermediate or poor nutritional value. This is consistent with population surveys conducted nationally and internationally. In Chile, adolescents (aged 14 years to 18 years) ranked first in the consumption of refined sugar (121 g/day) and second in the consumption of saturated fats (12.7 mL-g/day) compared to other age groups. In this age group, the consumption of sugar-sweetened soft drinks was 254 mL/day, according to the latest National Food Consumption Survey [53]. This survey also reported that 97% of children and adolescents aged 6 years to 18 years need to improve the quality of their diets. The World Health Organization’s Health Behaviour in School-aged Children (HBSC) survey found that in Scotland, 35% of adolescents eat sweets or chocolate every day, and 18% eat chips every day. In addition, 20% of Scottish female adolescents and 27% of their male counterparts consume sugary soft drinks daily [54]. Among US high schoolers, 22% of males and 17% of females report consumption of sugar-sweetened soft drinks ≥2 times per day [55].

### 4.2. Implications for Practice

Our results are of interest for a number of reasons. Translating research knowledge to practice and policy is much needed in the field of health promotion [56,57]. The idea of testing the connection between diet and cognition using functional cognitive measures such as GPA, graduation rates, and rates of taking college entrance exams was aimed at bridging the gap between research and policymaking. Although evidence on the consequences of unhealthy diets on learning and cognition is growing, the failure to implement effective interventions persists. A more informed approach to this connection can influence healthcare practitioners, educators and parents.

In addition, lower academic results have been associated with several health-risk behaviors in youths. In US adolescent populations, over the past three decades, cross-sectional and longitudinal studies demonstrate links connecting poor academic performance with sedentary lifestyle, alcohol/tobacco abuse, sexually risky behaviors, and violence [58]. All of these risk behaviors have been regarded as important contributors to poor health status in adulthood and multiple social problems. Since the influence of academic performance on future health is known [59], the relationship of diet and academic results may be an important public health tool. It is also important to identify the nutrients and dietary patterns that most influence cognitive health and academic performance.

The fact that adolescents struggle to make healthy dietary choices is not new information. Youthful anomalous health decision-making has been attributed to an aversion to forced choices; the inclination to rely on taste, brands, and convenience as primary drivers of food decisions; and the tendency to discount the value of delayed rewards or penalties [60]. Also, sufficient nutrition knowledge does not necessarily correspond to responsible dietary behavior [61]. Thus, associating healthy dietary choices with school performance can perhaps enhance the value of healthy eating and boost motivation. After all, academic achievement, academic behavior, and academic performance are closely linked to expectations of better postsecondary opportunities and subsequent job status [62,63].

Our results that show an association between a healthy diet and improved cognitive and educational outcomes should be a matter of interest to support nutrition interventions designed for adolescents. To date, the majority of interventions that emphasize the relationship between diet type and cognition and academics have been designed for infants and young children [6,19,52], who are less independent in their food choices. For health promotion purposes, unhealthy dietary habits during adolescence are usually said to be related to early onset of cardiometabolic disorders, including high blood pressure, type-2 diabetes and coronary heart disease, while arguments based on the potential cognitive impact of diet are still lacking. We have seen that adolescents are also exposed to the detrimental cognitive effects of a diet high in saturated fats and refined sugars. Moreover, adolescence is a transitional period with subcortical regions associated with reward-seeking and emotion developing earlier than prefrontal control regions [64]. Greater emotional reactivity and sensitivity may in part explain unhealthy dietary habits among teenagers. Sociocultural changes, the need to fit in, food availability and the quest for independent decision-making also contribute to unhealthy food choices that are common during adolescence [65], making this period one of tremendous importance in terms of cognitive development.

A further implication of these findings is that they can potentially play a major role in health promotion by educational agencies and schools. Dietary habits that comport with food guidelines might help pave the way for students on the path to higher education. Chilean high school students perform far below the Organization for Economic Co-operation and Development (OECD) average in mathematics, reading and science, with less than 2% of 15-year-old scoring in the group of top performers [63]. Evidence shows that students who fail to reach baseline levels of performance in these areas have difficulties with academic readiness, persistence and higher education completion [66]. Nonetheless, 80% of Chilean parents expect their children to obtain a college degree [63].

### 4.3. Limitations and Strengths

This research provides results that support a connection between nutritious dietary intake and higher academic achievement. Given that most studies have been conducted in the developed world, one strength of this study is that it provides evidence that may be useful for countries undergoing nutritional and epidemiological transitions. Second, the use of a translational research approach to explore the diet–learning–cognition connection and provide applicable results is a positive contribution. Further, to our best knowledge, this is the first study to investigate the association of nutrition and academic achievement on HS students’ postsecondary education intentions.

Despite these strengths, several limitations persist that should be considered when interpreting these results. Our sample is not representative of the Chilean adolescent population, as it consisted of adolescents from low and middle SES families. However, data from these socioeconomic groups may be especially important: population-based surveys conducted in Chile show that the prevalence of unhealthy dietary habits, physical inactivity, and excess weight is higher in adolescents from low and middle SES families compared to adolescents from high SES families [53,67]. This means that students from low and middle SES families are more exposed to risk factors for difficulties related to progressing from high school to higher education. Encouraging healthy dietary habits and, in particular, intake of healthy snacks, might smooth the pathway to college. Second, although we accounted for the effect of important confounders (including parental education and family structure), we were not able to consider other key influences, such as family support-related variables, general motivational factors (e.g., achievement motivation), and students’ interests in specific subject areas, which may also impact their academic functioning. A third limitation is the cross-sectional nature of the study. Since data on snack quality for each participant was recorded only once, it would be difficult to infer the temporal association between this exposure and the academic outcomes. Thus, only association, and not causation, can be inferred from our study. While our results may be useful to inform new hypotheses, a more complex investigation, such as a longitudinal study or crossover intervention trial, should be conducted to test the temporality of these associations, i.e., that the exposure to Western-type food items precede academic difficulties. Finally, future studies should replicate and extend this analysis in other young populations.

## 5. Conclusions

Poor nutritional quality snacking at school and at home was associated with poor secondary school academic achievement and lower intention to enroll in higher education. Both types of snacking showed similar associations with these educational outcomes. These results may have important implications for the promotion of healthy lifestyles by educational agencies and schools. Also, associating healthy snacking with educational outcomes can perhaps enhance the value of having responsible health behaviors and boost motivation for a healthy way of life.

## Figures and Tables

**Table 1:** Descriptive statistics of the sample: adolescent students from Santiago, Chile (*n* = 678).

Variables	Mean or *n*	SD or Percentage
Chronological age		
Age (years)	16.8	0.3
In-home snacking		
Healthy	180	26.55
Unhealthy-to-fair	337	49.71
Unhealthy	161	23.74
At-school snacking		
Healthy	183	26.99
Unhealthy-to-fair	302	44.54
Unhealthy	193	28.47
Academic outcomes		
Graduated high school	571	84.09
Took college admission exams * (*n* = 571)	387	67.76
High school GPA (score) (*n* = 571)	481.1	92.3
Sex		
Male	357	52.58
Anthropometrics		
BMI (z-score)	0.65	1.2
Weight status		
*Normal*	417	61.42
*Overweight*	167	24.59
*Obesity*	95	14.99
Physical activity		
Weekly scheduled PA ≤ 90 min	403	59.35
Parental education		
Maternal education: incomplete secondary	240	35.40
Paternal education: incomplete secondary	192	28.32
Family structure		
Fatherless family	274	40.4
Iron supplementation in infancy		
No added Fe (6–12 months)	286	42.18

* Only those students graduating from high school (*n* = 571) are allowed to take the exams for college admission. BMI: Body-Mass Index. Normal weight: BMI-z from −1 SD to +1 SD. Overweight: BMI-z from >1 SD to 2 SD. Obesity: BMI-z ≥ 2 SD. GPA: grade point average; SD: standard deviation; PA: physical activity.

**Table 2:** Estimated cross-sectional association between achieving the high school diploma (outcomes) and nutritional quality of in-home and at-school snacking (exposure) in students from Santiago, Chile, after adjusting other influences (*n* = 678).

	In-Home Snacking				At-School Snacking			
	OR	95% CI	aOR	95% CI	OR	95% CI	aOR	95% CI
Unhealthy	0.44 **	0.25–0.82	0.47 *	0.25–0.88	0.47 *	0.26–0.83	0.49 *	0.27–0.89
Unhealthy-to-fair	0.67	0.39–1.16	0.70	0.39–1.24	0.65	0.37–1.13	0.67	0.37–1.20
Male	(…)	-	0.42 ***	0.27–0.67	(…)	-	0.43 ***	0.27–0.68
Overweight	(…)	-	0.88	0.52–1.46	(…)	-	0.89	0.53–1.48
Obesity	(…)	-	0.81	0.44–1.49	(…)	-	0.81	0.44–1.49
Physically inactive	(…)	-	0.37 ***	0.22–0.61	(…)	-	0.37 ***	0.22–0.63
Maternal education	(…)	-	0.66	0.42–1.02	(…)	-	0.66	0.42–1.02
Paternal education	(…)	-	0.91	0.55–1.47	(…)	-	0.91	0.56–1.48
Fatherless family	(…)	-	0.77	0.51–1.20	(…)	-	0.77	0.50–1.19
No added Fe	(…)	-	0.89	0.57–1.37	(…)	-	0.89	0.58–1.38

OR: Odds ratio. aOR: adjusted OR. (…) Non-observed variables. Overweight: BMI-z from >1 SD to <2 SD. Obesity: BMI-z ≥ 2 SD. Physically inactive: ≤90 min/week of scheduled exercise. Maternal and paternal education: incomplete high school. * *p* < 0.05; ** *p* < 0.01; *** *p* < 0.001.

**Table 3:** Estimated cross-sectional association between taking the exams for higher education (outcome) and nutritional quality of in-home and at-school snacking (exposure) in students from Santiago, Chile, after adjusting other influences (*n* = 571).

	In-Home Snacking				At-School Snacking			
	OR	95% CI	aOR	95% CI	OR	95% CI	aOR	95% CI
Unhealthy	0.46 **	0.29–0.71	0.53 *	0.31–0.88	0.49 ***	0.32–0.74	0.57 *	0.35–0.90
Unhealthy-to-fair	0.68 *	0.47–0.98	0.75	0.48–1.15	0.71	0.49–1.04	0.81	0.51–1.27
Male	(…)	-	0.66 *	0.45–0.96	(…)	-	0.66 *	0.45–0.97
Overweight	(…)	-	0.99	0.64–1.52	(…)	-	0.99	0.65–1.55
Obesity	(…)	-	0.97	0.56–1.66	(…)	-	0.97	0.57–1.67
Physically inactive	(…)	-	0.85	0.57–1.25	(…)	-	0.84	0.57–1.24
Maternal education	(…)	-	0.63 *	0.42–0.92	(…)	-	0.63 *	0.42–0.92
Paternal education	(…)	-	0.75	0.49–1.13	(…)	-	0.76	0.50–1.15
Fatherless family	(…)	-	0.68 *	0.48–0.99	(…)	-	0.68 *	0.47–0.98
No added Fe	(…)	-	0.84	0.59–1.21	(…)	-	0.84	0.58–1.21

OR: Odds ratio. aOR: adjusted OR. (…) Non-observed variables. Overweight: BMI-z from >1 SD to <2 SD. Obesity: BMI-z ≥ 2 SD. Physically inactive: ≤90 min/week of scheduled exercise. Maternal and paternal education: incomplete high school. * *p* < 0.05; ** *p* < 0.01; *** *p* < 0.001.

**Table 4:** Cross-sectional association between academic attainment in high school (outcome) and nutritional quality of in-home snacking and at-school (exposure) in students from Santiago, Chile, after adjusting other influences (*n* = 571).

	In-Home Snacking			At-School Snacking		
Mean GPA	Mean	Mean		Mean	SD	
Unhealthy (1)	490.0	473.2		473.2	90.2	
Unhealthy-to-fair (2)	502.2	486.8		486.8	89.3	
Healthy (3)	530.1	512.4		512.4	93.6	
Comparison of mean GPA ^§^	Mean diff.	95% CI	*d* ^ǂ^	Mean diff.	95% CI	*d* ^ǂ^
(1) vs. (2)	−12.2	−32.7; 4.6	0.09	−13.6	−33.2; 2.4	0.15
(1) vs. (3)	−40.1 ***	−59.2; −16.9	0.41	−39.2 ***	−57.0; −17.1	0.44
(2) vs. (3)	−27.9 ***	−43.5; −8.2	0.30	−25.6 *	−40.6; −4.9	0.31

GPA: Grade point average (expressed in score according to the Ministry of Education). ^§^ ANCOVA: * *p* < 0.05; *** *p* < 0.001. Models were adjusted for sex, weight status, familial background and iron supplementation in infancy. ^ǂ^ Cohen’s *d* coefficients account for the effect of different sample sizes. ESs around 0.20 are of policy interest when they are based on measures of academic achievement [33]. ANCOVA: analysis of covariance.
